# Identification and analysis of the secretome of plant pathogenic fungi reveals lifestyle adaptation

**DOI:** 10.3389/fmicb.2023.1171618

**Published:** 2023-04-20

**Authors:** Mingxuan Jia, Xiaodong Gong, Mengmeng Fan, Haoran Liu, He Zhou, Shouqin Gu, Yuwei Liu, Jingao Dong

**Affiliations:** ^1^State Key Laboratory of North China Crop Improvement and Regulation, Baoding, China; ^2^College of Life Sciences, Hebei Agricultural University, Baoding, China; ^3^Hebei Bioinformatic Utilization and Technological Innovation Center for Agricultural Microbes, Baoding, China; ^4^College of Plant Protection, Hebei Agricultural University, Baoding, China

**Keywords:** fungi, secretome, effector, plant pathogens, saprotrophs, symbionts

## Abstract

The secretory proteome plays an important role in the pathogenesis of phytopathogenic fungi. However, the relationship between the large-scale secretome of phytopathogenic fungi and their lifestyle is not fully understood. In the present study, the secretomes of 150 plant pathogenic fungi were predicted and the characteristics associated with different lifestyles were investigated. In total, 94,974 secreted proteins (SPs) were predicted from these fungi. The number of the SPs ranged from 64 to 1,662. Among these fungi, hemibiotrophic fungi had the highest number (average of 970) and proportion (7.1%) of SPs. Functional annotation showed that hemibiotrophic and necrotroph fungi, differ from biotrophic and symbiotic fungi, contained much more carbohydrate enzymes, especially polysaccharide lyases and carbohydrate esterases. Furthermore, the core and lifestyle-specific SPs orthogroups were identified. Twenty-seven core orthogroups contained 16% of the total SPs and their motif function annotation was represented by serine carboxypeptidase, carboxylesterase and asparaginase. In contrast, 97 lifestyle-specific orthogroups contained only 1% of the total SPs, with diverse functions such as PAN_AP in hemibiotroph-specific and flavin monooxygenases in necrotroph-specific. Moreover, obligate biotrophic fungi had the largest number of effectors (average of 150), followed by hemibiotrophic fungi (average of 120). Among these effectors, 4,155 had known functional annotation and pectin lyase had the highest proportion in the functionally annotated effectors. In addition, 32 sets of RNA-Seq data on pathogen-host interactions were collected and the expression levels of SPs were higher than that of non-SPs, and the expression level of effector genes was higher in biotrophic and hemibiotrophic fungi than in necrotrophic fungi, while secretase genes were highly expressed in necrotrophic fungi. Finally, the secretory activity of five predicted SPs from *Setosphearia turcica* was experimentally verified. In conclusion, our results provide a foundation for the study of pathogen-host interaction and help us to understand the fungal lifestyle adaptation.

## Introduction

Secretome is a group of proteins synthesized in the cell and secreted to extracellular to function in eucaryotes (McCotter et al., 2016). Most secreted proteins (SP) are secreted through the classic endoplasmic reticulum (ER) and Golgi pathway (Rapoport et al., 2017; Pantazopoulou and Glick, 2019). A few proteins are secreted through nonclassical secretory pathways due to the lack of post-translational modification of the ER or Golgi (He et al., 2021). In terms of functions, fungal secretome can be divided into two classes. One is the SPs with enzyme functions, and the other is not, such as stress-related proteins.

During plant pathogenic fungi infecting the host plant, fungi have evolved complementary strategies to overcome host resistance. The cuticle is the first barrier for fungi to break through the plant epidermis. Plant pathogenic fungus catalyzes the hydrolysis of cutin polymers on the host surface by secreting cutinase (Morais Do Amaral et al., 2012). Second, many fungi decompose pectin, cellulose, and other substances in the plant cell wall by secreting cell-wall degrading enzymes and absorbing polysaccharides for their growth and reproduction (Ospina-Giraldo et al., 2010). Third, some fungus secretes serine-rich toxins to inhibit the activity of defensive enzymes and reduce the vitality of plant cells (Muszewska et al., 2017). Toxins can also bind to cell membranes, altering their physiological function and causing necrosis of vascular cells, leading to plant wilt. Importantly, secretory proteins are involved in the formation of infected structures. For example, in *Magnaporthe grisea*, the expression of cutinase 2 is significantly up-regulated during the formation and maturation of appressorium, which plays a crucial role in the formation of penetration peg (Skamnioti and Gurr, 2007). In addition, during the interaction between plant pathogenic fungi and their host, SPs play an important role in pathogenic processes such as invasion and colonization (Doehlemann et al., 2009). Some small SPs have been identified as effectors, which are cysteine-rich proteins and play a role in virulence (Lo Presti et al., 2015). Effectors can suppress pattern-triggered immunity (PTI), thus allowing the pathogen to successfully infect the host. This molecular mechanism is considered to be the core mechanism of interaction between fungi and host (Jones and Dangl, 2006). Plant pathogenic fungi have diverse lifestyles, including obligate and facultative biotrophic, hemibiotrophic, necrotrophic, saprophytic, and symbiotic, in which they develop different strategies to interact with their host plants. Several studies have reported that some components of the fungal secretome are associated with its lifestyle (Lowe and Howlett, 2012; Kim et al., 2016), such as the small SPs and effectors. However, the relationship between the total secretome and lifestyle, as well as the expression pattern of the secretome during fungal infection, is not fully understood.

In recent years, the increasing number of sequenced fungal genomes and the availability of transcriptomic data on pathogen-host interactions, as well as the updating of secretory protein prediction software, have provided the basis for further elucidation of secretome and lifestyle adaptations. In this study, we developed a multi-procedure data extraction script to predict the SPs of 150 fungi. Furthermore, the function of these SPs were classified, and the differences in the composition and function of the SPs between fungi with different lifestyles were compared. The core-like SPs and lifestyle-specific SPs were identified. In addition, the expression level of SP genes and non-SP genes with different functions were studied. Our studies will provide a comprehensive understanding of fungal-SPs in different fungi and lifestyles and lay a foundation for the function research of fungal secretome.

## Materials and methods

### Prediction of SPs and effect-like proteins

Proteome sequences of 150 fungi were obtained from the Joint Genome Institute, NCBI, and Genome Projects at University of Kentucky (Supplementary Table S1). SignalP 5.0 was used to predict the sequence containing the signal peptide at the N-terminal of the protein (prediction = SP; Almagro Armenteros et al., 2019b). TMHMM 2.0b was used to predict 0 or 1 transmembrane domain proteins (number of predicted TMHs = 0/1; Krogh et al., 2001). Phobius Ver 1.01 was used to reduce false positives in SignalP analysis of signal peptides and transmembrane domains (SP = Y; Käll et al., 2007). WoLF PSORT V0.2 (best hit: extr) was used to predict SPs whose subcellular location is extracellular (Horton et al., 2007). TargetP 2.0 was used to filter chloroplast transit peptide (CTP) and mitochondrial targeting peptide (MTP) at the N-terminal of proteins (Almagro Armenteros et al., 2019a). PROSITE Scan was used to filter the proteins containing the endoplasmic reticulum retention signal (ProSite: PS00014). PredGPI was used to predict proteins without glycosylphosphatidylinositol (GPI) sites (specificity <99.5; Sigrist et al., 2010). The effector-like proteins in the secretome were then predicted. First, a Perl script was used to analyze the cysteine content and amino acid residue base of the predicted SPs. We selected proteins with a cysteine content greater than 3% and an amino acid residue number less than 400 as candidate effectors. Finally, effectorP 1.0 and effectorP 2.0 were used to predict effectors, and the proteins predicted by both software were considered as effectors (Sperschneider et al., 2016, 2018).

### Functional annotation of SPs

To obtain the GO annotation and Pfam domain annotation of the SP, the InterProScan-5.47-82.0 was used to annotate the predicted secretome (Quevillon et al., 2005). HMMER 3.1B2 was used to identify carbohydrate enzymes in the secretion group (E-value < 1e-5 and selection of the best hit; Eddy, 2009). The sources of the carbohydrate enzyme datasets were from dbCAN2.^1^ BlastP 2.7.1 was used to identify proteases and peroxidases in the secretome (*p*-value <1E-05 and selection of the best hit; Altschul et al., 1990). The protease and peroxidase datasets were downloaded from MEROPS^2^ and fPoxDB,^3^ respectively.

### Orthologous clustering of SPs

To reduce the influence of different strains of the same species on the homology analysis, 141 fungi were selected for further analysis. Cluster analysis of SPs was performed using Orthorfinder with default parameters, and only orthogroups containing at least five different species were considered for further analysis (Emms and Kelly, 2019). Clusters were defined as follows: (1) Core group: Gene clusters containing at least 80% of the species members. (2) Biotrophic specific group: Gene clusters containing only biotrophic members. (3) Necrotrophic specific group: Gene clusters containing only necrotrophic members. (4) Hemibiotrophic specific group: gene clusters containing only hemibiotrophic members. (5) Saprophytic specific group: gene clusters containing only saprophytic members. (6) Symbiotic specific group: gene clusters containing only symbiotic members. The results of the cluster analysis were shown using jvenn to generate Venn diagrams.

### Expression analysis of SP genes

RNA-seq data of plant-pathogen interactions from 32 species were obtained from NCBI, and the genome files and annotation files were obtained from the Joint Genome Institute, and detailed information were listed in Supplementary Table S2. HISAT2 software was used to map clean reads to the fungal genome, and StringTie was used to calculate the TPM of genes (Pertea et al., 2016). Subsequently, datasets of three hemibiotrophic fungi (SRP062877, SRP192548, and SRP069885) and two biotrophic fungi (SRP038019 and SRP117697) were selected for further analysis. Genes with TPM > 1 at least in one stage were counted. The TBtools was used to plot the heatmaps (Chen et al., 2020).

### Validation of secretory activity of the signal peptide

The signal peptide secretory activity of five SPs from *Setosphearia turcica* was verified using a yeast sucrase deletion mutant (YTK12). The signal peptide sequences of the candidate proteins were amplified by PCR using primers listed in Supplementary Table S3. The signal peptide sequences were cloned into the yeast vector pSUC2 with the *EcoR I*/*Xho I* restriction site. The pSUC2-signal peptide vector, the negative control vector pSUC2-Mg87^SP^ (Jacobs et al., 1997; Fang et al., 2016), and the positive control vector pSUC2-Avr1b were then transformed into YTK12, respectively. Sucrase from YTK12 which carrying a signal peptide was secreted extracellularly and hydrolyzed sucrose into monosaccharides, which then monosaccharides reacted with 2,3,5-triphenyltetrazolium chloride (TTC) to produce a çred triphenyl tetrazolium chloride precipitate.

## Results

### Identified of the secretome of plant pathogenic fungi

**Figure 1:**
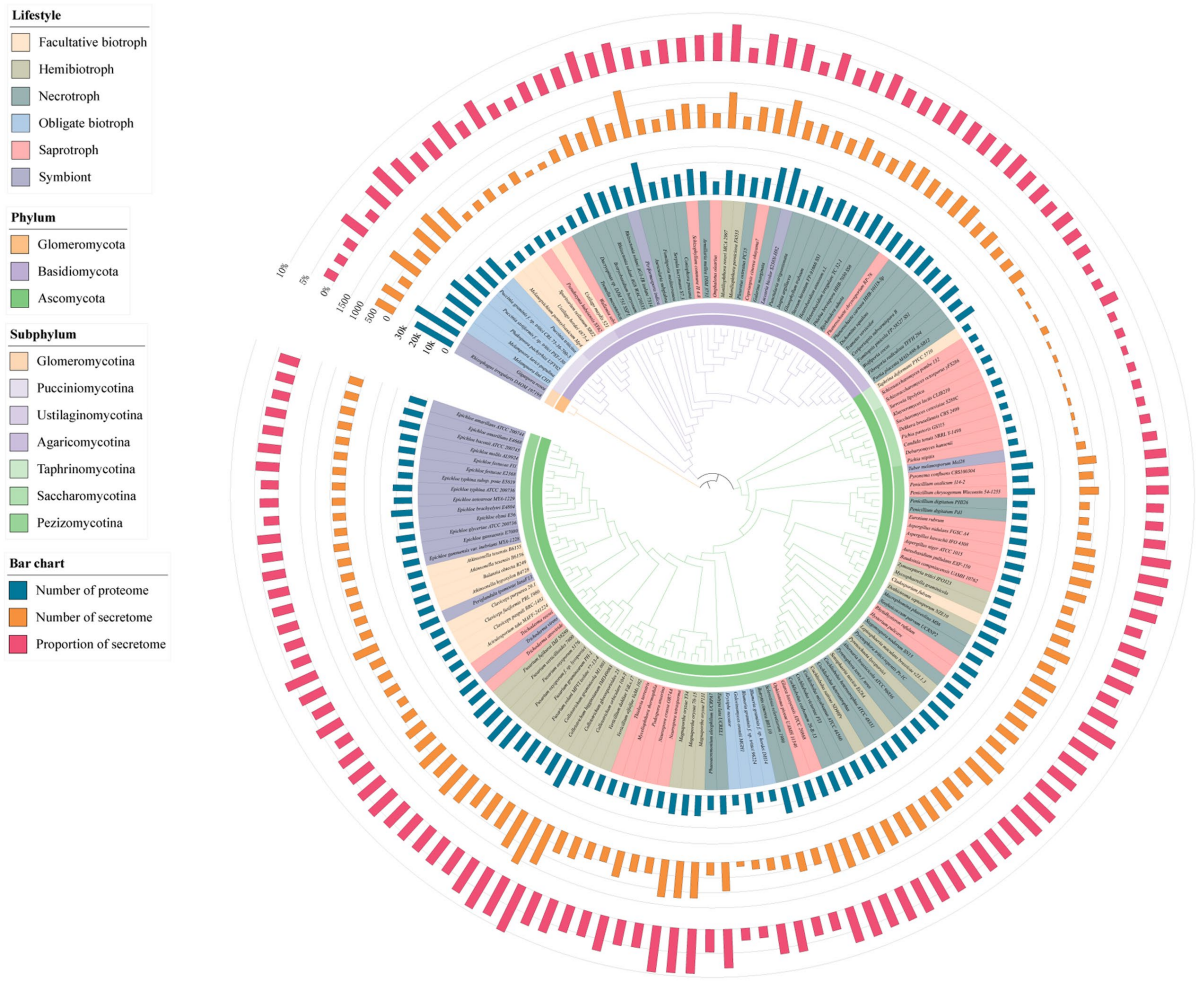
Species phylogenetic tree of 150 fungi and the number of proteome, number of secretome and proportion of secretome. The inner ring color of the evolutionary tree represents the phylum and subphylum classification of the fungi. The color in the species name represents the lifestyle of the fungi. The bar chart on the outer side of the evolutionary tree represents the proteome size, secretome size and the percentage of secreted proteins (SP).

To analyze the characteristics of the fungal secretome, the proteomic sequences from 150 fungi were used to identify the classical SPs which contain the signal peptide. These fungi covered the phyla including ascomycota, basidiomycota, glomeromycotan. The lifestyles of these fungi were represented by biotroph, hemibiotroph, necrotroph, symbiont, and saprotroph (Supplementary Table S1). The biotroph was further divided into facultative biotroph and obligate biotroph (Figure 1). Using the custom pipeline (Supplementary Figure S1), 94,974 SPs were predicted from 1,815,294 proteins of the 150 fungi, with an average of 633 SPs in each fungus. The number of SPs ranged from 64 of the saprotrophic fungal *Dekkera bruxellensis* to 1,662 of the obligate biotrophic fungus *Colletotrichum gloeosporioides*. Moreover, the secretome accounted for an average of 5.2% of the total proteins, the lowest was 1.1% of *D. bruxellensis*, and the highest was 10.4% of the hemibiotrophic fungus *Colletotrichum orbiculare*. To further explore the relationship between the secretome and their lifestyles, the amount and the proportion of SPs in fungi with different lifestyles were compared. We found that the hemibiotrophic fungi contained the most SPs (967 on average), such as *Magnaporthe oryzae* (1,245), *Colletotrichum higginsianum* (1,359), *C. orbiculare* (1,396), and *Colletotrichum gloeosporioides* (1,662). In addition, the number of SPs in necrotrophic fungi (737 on average) and obligate biotrophic fungi (650 on average) was approximate, respectively. However, facultative biotrophic fungi had the fewest SPs (414 on average) and the number was less than those in symbiontic fungi (516 on average) and saprotrophic fungi (481 on average). These results suggested that fungi with more complex lifestyles, such as hemibiotrophic fungi which have an initial biotrophic life-style and a subsequent necrotrophic phase, have more SPs.

### Diverse functions of SPs in fungi

To understand the functions of the SPs, the InterPro database was used to scan the GO annotation of them. The annotation results showed that 35.4% of the SPs had at least one GO term annotation. Because the SPs belonged to extracellular proteins, the molecular functions and biological processes of the GO terms were further studied. In the term of biological process, the most of annotated GO terms were presented by carbohydrate metabolic process (GO:0005975), proteolysis (GO:0006508), obsolete oxidation–reduction process (GO:0055114), organic substance metabolic process (GO:0071704), and lipid metabolic process (GO:0006629). For the molecular function part, the most common GO terms were hydrolase activity, hydrolyzing O-glycosyl compounds (GO:0004553), oxidoreductase activity (GO:0016491), flavin glands Purine dinucleotide binding (GO:0050660), serine-type peptidase activity (GO:0008236), and hydrolase activity (GO:0016787; Supplementary Figure S2). These results indicated that a considerable number of SPs had enzymatic activity. Furthermore, to explore the relationship between the functions of SPs and lifestyles, the enzymes with the most GO terms, namely carbohydrate-active enzymes (CAZymes), proteases and peroxidases were analyzed. On average, 32% of all of the SPs belonged to the three types of enzymes mentioned above, in which CAZymes were the most, followed by proteases and peroxidases.

**Figure 2:**
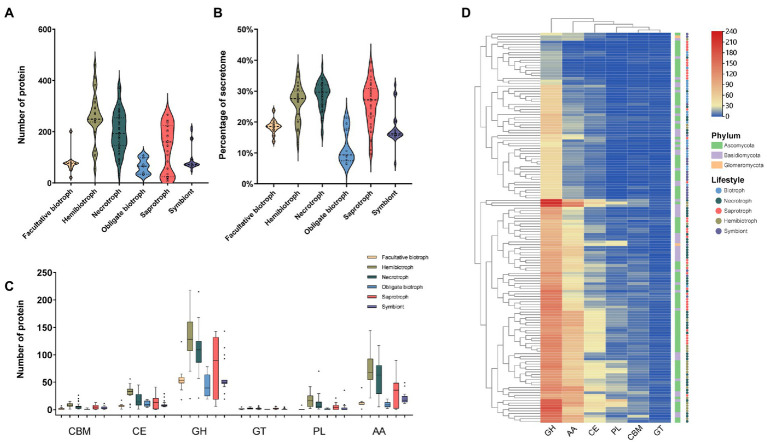
The number, proportion, family and clustering heat map of CAZyme in SPs. **(A)** Number of CAZyme in fungi with different lifestyles. **(B)** Proportion of CAZyme in fungi with different lifestyles to the secretome. **(C)** Number of members of different CAZyme families (glycoside hydrolases: GH, glycosyltransferases: GT, polysaccharide lyases: PL, carbohydrate esterases: CE, carbohydrate binding modules: CBM, auxiliary activities: AA). **(D)** Clustering heat map of CAZymes, with the square legend indicating the phylum to which the fungus belongs and the circle legend indicating the fungal lifestyle.

Previous research indicated that CAZymes are involved in the degradation of the plant cell wall and promote attachment, invasion, colonization, and nutrient acquisition from the hosts (Ospina-Giraldo et al., 2010; Barrett et al., 2020). In the present research, we found that the number of CAZymes distributed widely in different fungi of the same lifestyle and the number of CAZymes in different lifestyle fungi varies greatly. Specifically, hemibiotrophic fungi encoded the most CAZymes, with an average of 259, followed by necrotrophic, saprotrophic, symbiontic, facultative biotrophic, and obligate biotrophic fungi with an average of 200, 138, 95, 78, and 65, respectively (Figure 2A). Further, the proportion of fungal CAZymes in SPs from the fungi with different lifestyles showed significantly different. The CAZymes accounted for more than 25% of SPs in necrotrophic (28%), hemibiotrophic (26%), and saprotrophic (26%) fungi. The proportion of CAZymes in symbiontic and facultative biotrophic fungi were similar with the proportion of 18 and 17% and that in obligate biotrophic fungi were the lowest (13%; Figure 2B). Further, software hmmsearch was used to align the SPs to the datasets of dbCAN2, in which CAZymes were divided into six categories: glycoside hydrolases (GH), glycosyltransferases (GT), polysaccharide lyases (PL), carbohydrate esterases (CE), carbohydrate binding modules (CBM), auxiliary activities (AA; Figure 2C). Among them, the GH family was the most abundant in our research. In detail, the hemibiotrophic fungi encoded the most GH family (an average of 129), such as *C. gloeosporioides* and *Moniliophthora roreri* contained more than 400 GH enzymes, respectively. The necrotrophic fungi also had a high level of GH enzymes (109 on average), the saprotrophic fungi had an average of 80 GH enzymes, symbiontic and biotrophic fungi had less GH enzymes, which number was 60 and 51 on average. However, there were some exceptions in symbiontic fungi and biotrophic fungi, such as *Cladosporium fulvum* and *Periglandula ipomoeae* contained more than 100 GH enzymes, respectively. Moreover, the number of AA enzymes in hemibiotrophic and necrotrophic fungi was higher than that in other lifestyles, with an average of 71 and 56, respectively. Saprotrophic fungi contained an average of 33 AA enzymes. The number of AA enzymes in biotrophic and symbiontic fungi was only 20 and 11 on average. The CE family was widely distributed among the studied fungi, only 5 saprophytic fungi did not encode this family protein. The number of CE families in hemibiotrophic fungi (an average of 31) was significantly higher than that in other lifestyles (an average of 13). The PL family was more abundant in fungi in hemibiotrophic and necrotrophic fungi, but rarely in biotrophic and symbiotic fungi. In addition, cluster analysis of the CAZyme family revealed significant expansion of GH, PL, CE, and AA families in necrotrophic and hemibiotrophic fungi (Figure 2D). These results suggest that fungi with a necrotrophic phase need to kill host plant cells by secreting large amounts of cell wall degrading enzymes, of which GH, PL, CE, and AA may play a major function.

**Figure 3:**
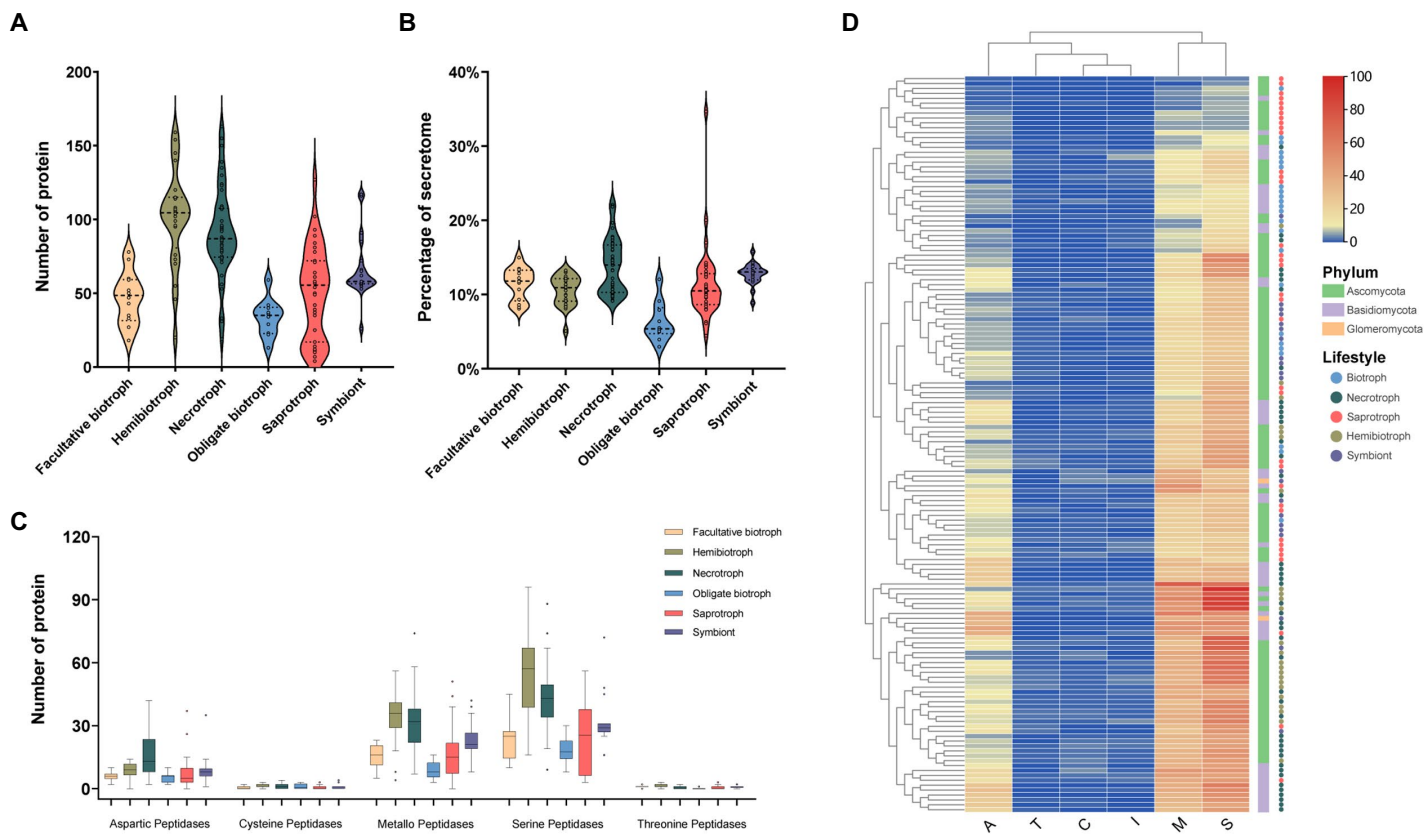
The number, proportion, family and clustering heatmap of protease in SPs. **(A)** Number of protease in fungi with different lifestyles. **(B)** Proportion of protease in fungi with different lifestyles to the secretome. **(C)** Number of members of different protease families. **(D)** Clustering heat map of protease, with the square legend indicating the phylum to which the fungus belongs and the circle legend indicating the fungal lifestyle.

Protease plays a vital role in the decomposition, assimilating nutrients, and attacking the plant host defense system in fungi (Muszewska et al., 2017). In our research, we found that the proteases accounted for about 7% of the secretome. The number of proteases ranged from 4 in the saprophytic fungus *Schizosaccharomyces pombe* to 163 in the necrotrophic fungus *R. solani*. Hemibiotrophs had the most secreted proteases (average 101), followed by necrotrophic, saprophytic, symbiotic, facultative and obligate biotrophic fungi with an average of 91, 63, 50, 46, and 38, respectively (Figure 3A). In terms of proportion, the proteases from necrotrophic fungi was the highest (13%), while the obligate biotrophic fungi was the lowest (6%; Figure 3B). This result suggest that the reduction of proteases in biotrophic fungi may be related to the fact that it is not necessary to kill host cells during the infection stage. Moreover, the most abundant protease families in most fungi were the serine protease family, followed by the metallopeptidase family and the aspartic peptidase family (Figure 3C). Cysteine peptidase and threonine peptidase were less, and many saprophytic and biotrophic fungi did not encode these two enzymes. The family cluster analysis of secreted proteases showed that some hemibiotrophic and all necrotrophic fungi clustered together indicating that they have a similar protease composition (Figure 3D).

**Figure 4:**
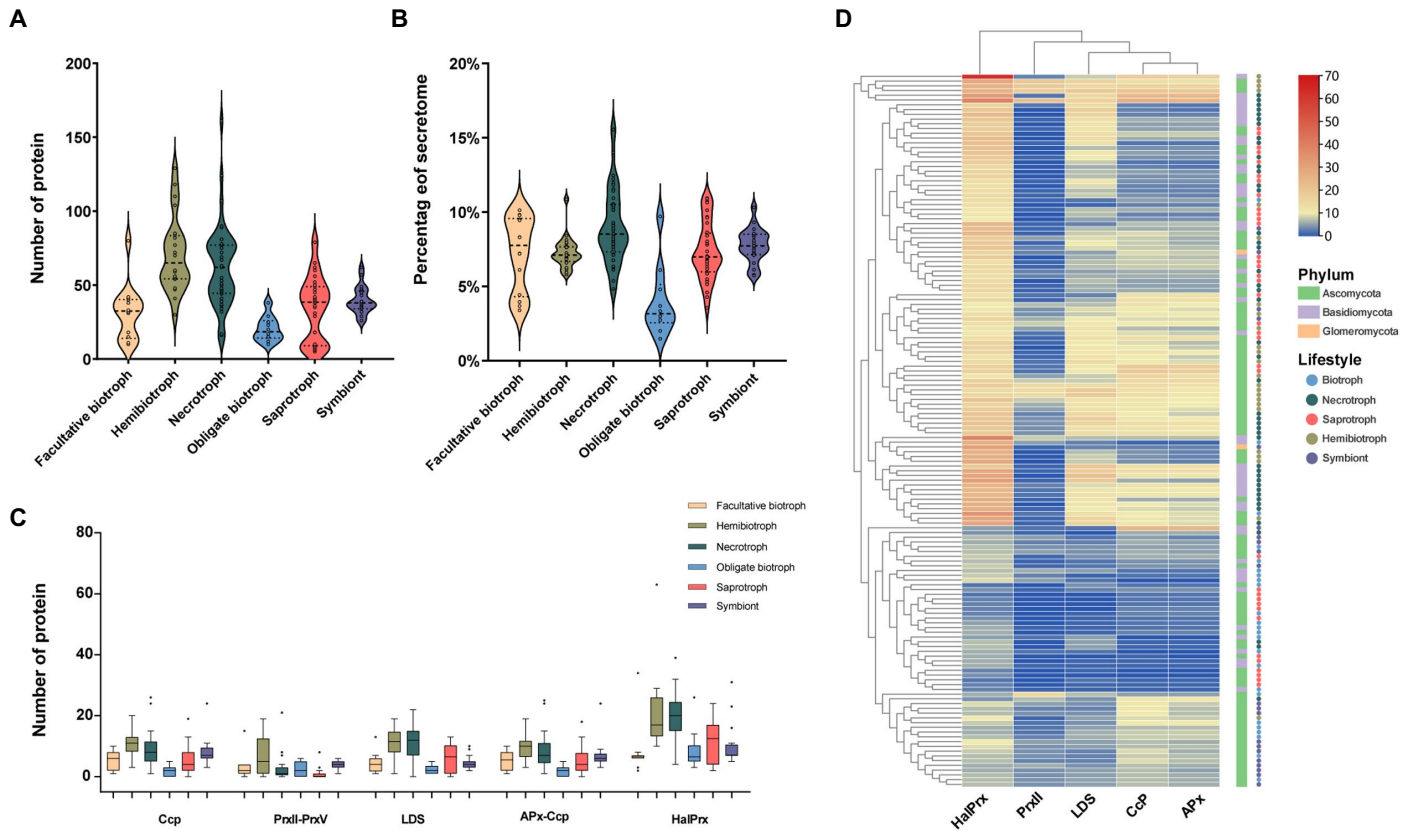
The number, proportion, family and clustering heatmap of peroxidase in SPs. **(A)** Number of peroxidase in fungi with different lifestyles. **(B)** Proportion of peroxidase in fungi with different lifestyles to the secretome. **(C)** Number of members of different peroxidase families (haloperoxidase: HalPrx, linoleate diol synthase: LDS, cytochrome: CcP, hybrid ascorbate-cytochrome c peroxidase: APx-Ccp, atypical 2-cysteine peroxiredoxin: PrxII) **(D)** Clustering heat map of peroxidase, with the square legend indicating the phylum to which the fungus belongs and the circle legend indicating the fungal lifestyle.

Peroxidase is belonged to oxidoreductase and plays an essential role in the pathogenicity and fixation of carbon sources in fungi (Hemetsberger et al., 2012; Choi et al., 2014; Mir et al., 2015). Our results showed that the peroxidase accounted for about 5% of the secretome and the number of the enzymes ranged from 5 in saprophytic fungal *Debaryomyces hansenii* to 155 in necrotrophic fungal *Auricularia subglabra*. Similar to the distribution of CAZymes and protease, hemibiotrophic and necrotrophic fungi contained the most peroxidases, 70 and 62, respectively, followed by symbiontic (39), saprotrophic (35), facultative biotrophic (31), and obligate biotrophic fungi (20; Figure 4A). In terms of proportion, the peroxidease from necrotrophic fungi was the highest (9%), while the obligate biotrophic fungi was the lowest (3.9%; Figure 4B). Further analysis showed that haloperoxidase (HalPrx), linoleate diol synthase (LDS), cytochrome (CcP), hybrid ascorbate-cytochrome C peroxidase (APx), atypical 2-cysteine peroxiredoxin (PrxII), and class II peroxidase (CII) were ubiquitous peroxidase in these fungi (Figure 4C). Cluster analysis of peroxidase family showed that the biotrophic fungi and symbiotic fungi had similar peroxidase composition, and the hemibiotrophic fungi and necrotrophic fungi had similar peroxidase composition, but the peroxidase composition of saprophytic fungi did not show obvious rule (Figure 4D). In addition, other peroxidases rarely presented in these fungi, for instance, obligate biotrophic fungi contained a small number of peroxidase families and did not code the manganese peroxidase (MnP), respiratory burst oxidase (Rbohs), and fungi-bacteria glutathione peroxidase (GPx) families. Furthermore, the MnP family, which are the essential enzymes for attacking lignocellulose, was only found in six wood-decay necrotrophic fungi.

### Identification and functional annotation of effectors in SPs

**Figure 5:**
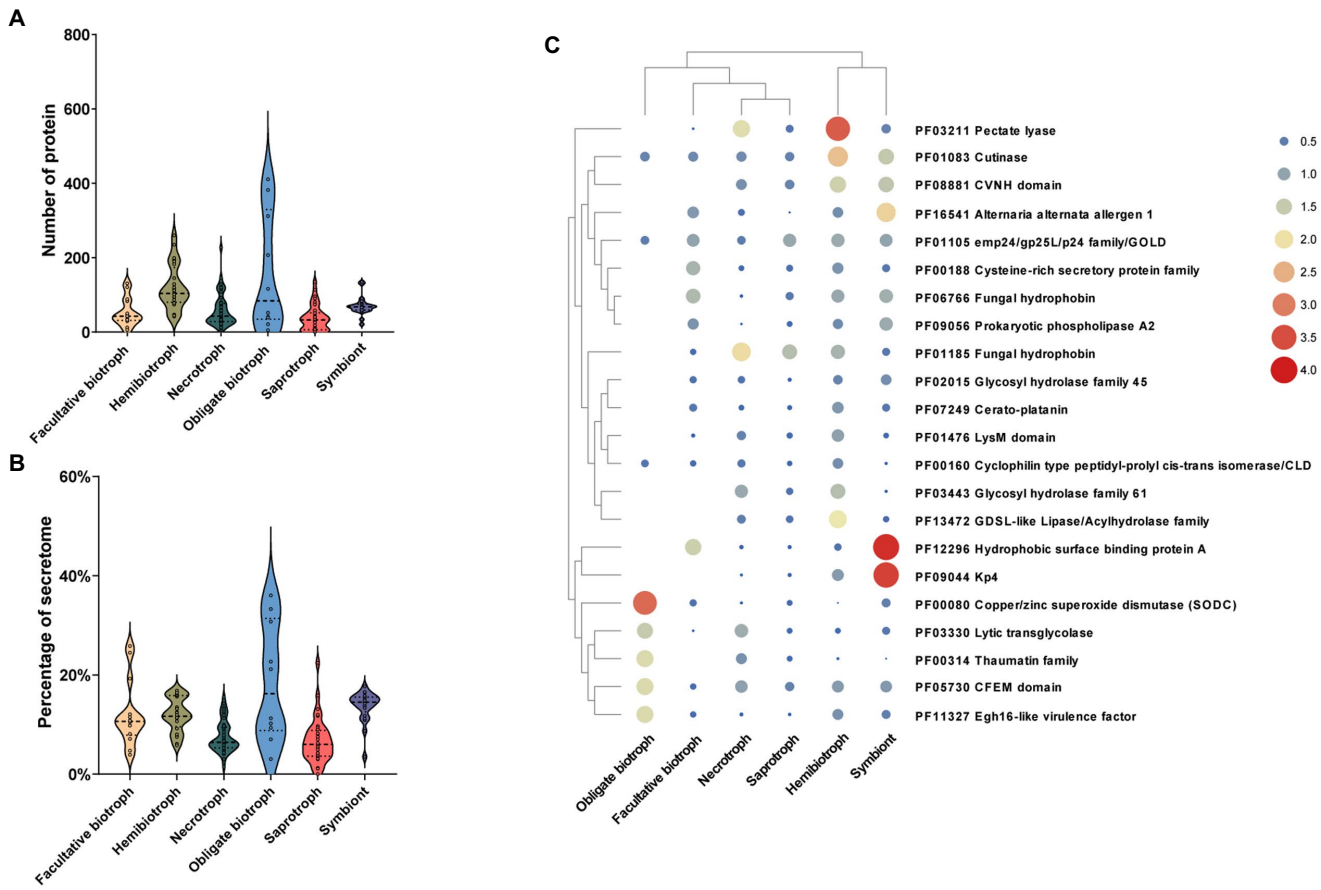
Relationship between the number, ratio, function of effector factors and fungal lifestyle. **(A)** Number of effector in fungi with different lifestyles. **(B)** Proportion of effector to secretome in fungi with different lifestyles. **(C)** Clustering heat map of effector functions, the color and size of the circles represent the richness of this effector function.

The effector, as a small SP, inhibits the PTI response of hosts by regulating the transduction of defense signals and downstream responses (Lo Presti et al., 2015). In the present study, based on the characteristics of the proteins, including the number of amino acid residues and cysteine content, the effector-like proteins were filtered and further confirmed using effectorP software. The results showed that there was a significant difference in the number of effectors from different lifestyles. In detail, the number of effectors in obligate biotrophic fungi (average of 158) was the most than that in other lifestyles, followed by hemibiotrophic (average of 120), symbiontic (average of 120), necrotrophic (average of 58) and saprotrophic fungi (average of 37; Figure 5A). Further, we found that the number of effectors in obligate biotrophic fungi varied greatly. Such as *Melampsora larici-populina* (411), *Puccinia graminis* (382), and *Puccinia striiformis* (312) secreted the most effectors among all fungi, while, only one effector is found in obligate biotrophic fungus *Erysiphe Necator*. Although, facultative biotrophic fungi encoded a low number of effectors (54 on average), their effectors accounted for a high proportion of SPs (12%), higher than the saprophytic fungi (6%), necrotrophic fungi (7%) and similar to hemibiotrophic fungi (12%) and symbiotic fungi (13%; Figure 5B). These results indicated the fungi that whose survival depended much more on their living hosts have more effector than the fungi whose survival depended on their dead hosts.

To further explore the function of effectors in different lifestyle fungi, the Interpro database was used to functionally annotate the predicted effectors. Among the 10,858 effectors, only 35% (3,789) had annotations with 331 different Pfam terms. Further, 22 annotated Pfam terms that contained at least 50 effectors were analyzed further. The most functional annotation term which contains 189 effectors was pectin lyases, which abundantly existed in hemibiotrophic and necrotrophic fungi, less in saprotrophic fungi and symbiotic fungi, and rare in biotrophic fungi (Figure 5C). The second most presented annotation term was hydrophobins, a class of enzyme that regulate the communication between fungi and their environment, which is widespread in fungi and more abundant in Basidiomycetes than in other fungi. In addition, we found that cutinase effector was more abundant in hemibiotrophic and symbiotic fungi and less abundant in biotrophic, necrotrophic and saprophytic fungi, suggesting that hemibiotrophic and symbiotic fungi may require enhanced attachment of fungal spores to the plant surface. In addition, we found that some effectors were enriched only in certain lifestyle fungi. For example, hydrophobic surface binding protein A and KP4 effectors were enriched in symbiotic fungi, suggesting a symbiotic strategy whereby symbiotic fungi obtain nutrients from their hosts while suppressing competing fungi. These results indicating that the lifestyle-specific effectors may play an important role in their lifestyles.

### Identification conservation and lifestyle-specific SPs

**Figure 6:**
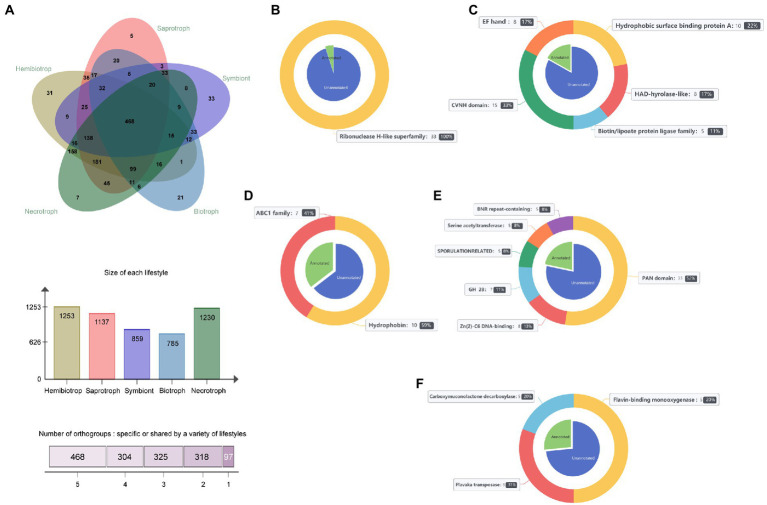
The orthogroups in 141 fungi and the function of lifestyle-specific orthogroups. **(A)** Overview of the number of orthogroups in 141 fungi. **(B)** Functions performed by symbiotic fungal-specific orthogroups. **(C)** Functions of the saprophytic fungal-specific orthogroups. **(D)** Functions of the necrotrophic fungal-specific orthogroups. **(E)** Functions of the hemibiotrophic fungal-specific orthogroups. **(F)** Functions of the biotrophic fungal-specific orthogroups. The blue part of the pie chart on the inner side of the nested graph represents proteins without functional annotation. The green part represents proteins with functional annotations, and the bands on the outside of the nested plots indicate the different functions of lifestyle-specific proteins and the proportion of lifestyle-specific proteins that have that function.

To identify and distinguish SPs that play a crucial role in fungi with different lifestyles, the software OrthoFinder was used to cluster the secretome from 141 fungi. The results showed that 87,909 SPs were divided into 5,207 orthogroups, and the number of orthogroups ranging from 2 to 1,143. In addition, 7,226 SPs were not clustered with any other SPs and were named as unassigned genes. Subsequently, the core, shared and specific orthogroups in different lifestyles were then further investigated. The result showed that a total of 1,513 homologous groups were identified, of which 27 were core groups, 1,416 were lifestyle-shared groups and 468 were shared by all lifestyle fungi, and 97 were lifestyle-specific groups. Among of the homologous groups, hemibiotrophic fungi had the largest number of secretory protein groups with 1,253, necrotrophic fungi contained 1,230 homologous groups, saprophytic fungi contained 1,137 homologous groups, and symbiotic and biotrophic fungi contained fewer homologous groups with 859 and 758, respectively, (Figure 6A). Among them, there was only one core orthogroup containing all the fungal genes, and this orthogroup had a total of 889 members. The function of the proteins in this group was serine carboxypeptidase, while the other 26 core groups function mainly as aspartate proteases, multicopper oxidase, subtilase, etc. (Supplementary Table S4). In addition, we found that the fungi with close lifestyles tended to share more orthogroups (Figure 6A). For example, hemibiotrophic and necrotrophic fungi shared as many as 158 orthogroups, saprophytic and necrotrophic fungi shared 45 orthogroups, and biotrophic and symbiotic fungi shared 33 orthogroups. Further, the proteins of the orthogroup were annotated, and it was found that the lifestyle-specific group showed diverse functions (Figures 6B–F). There were 33 symbiotic fungal-specific orthogroups, including 273 proteins, of which 46 proteins were functionally annotated. Their functions mainly included the hydrophobic surface binding protein A, HAD-hyrolase-like, Biotin/lipoate A/B protein ligase family, chitin-binding protein (CVNH) and calcium ion buffering protein (EF-hand). Saprophytic fungi had only 5 specific orthogroups, including 48 proteins, of which 17 proteins had functional annotations, including fungal hydrophobins and ABC1 family protein kinases. Seven necrotrophic fungal-specific orthogroups were containing 98 SPs, 26 of which were functionally annotated, including flavin-binding monooxygenase, carboxymuconolactone decarboxylase, and plavaka transposase. The 31-specific orthogroups of hemibiotrophic fungi contained 227 SPs, of which 63 proteins were functionally annotated, including PAN/Apple domains contained proteins, Zn(2)-C6 fungal-type DNA-binding proteins, glycosyl hydrolases family 28 proteins, sporulation-related proteins, homoserine/serine acetyltransferase, and BNR repeat-containing family. Biotrophic fungi possessed 21 specific orthogroups, including 306 SPs, of which only 16 proteins were annotated and belonged to the Ribonuclease H-like superfamily. Further analysis of biotrophic fungi-specific homologous groups revealed that there were 290 proteins with no functional annotation, of which 155 were effectors, suggesting that these lifestyle specific effectors may be related to the adaption of their lifestyles. In summary, the above functional annotation of these orthogroups revealed significant differences in gene function among fungal orthogroups with different lifestyles, and these differences in function may be appropriate for the specific lifestyle of the fungi.

### Exploration the role of different components of secretome in infection using RNA-Seq data

**Figure 7:**
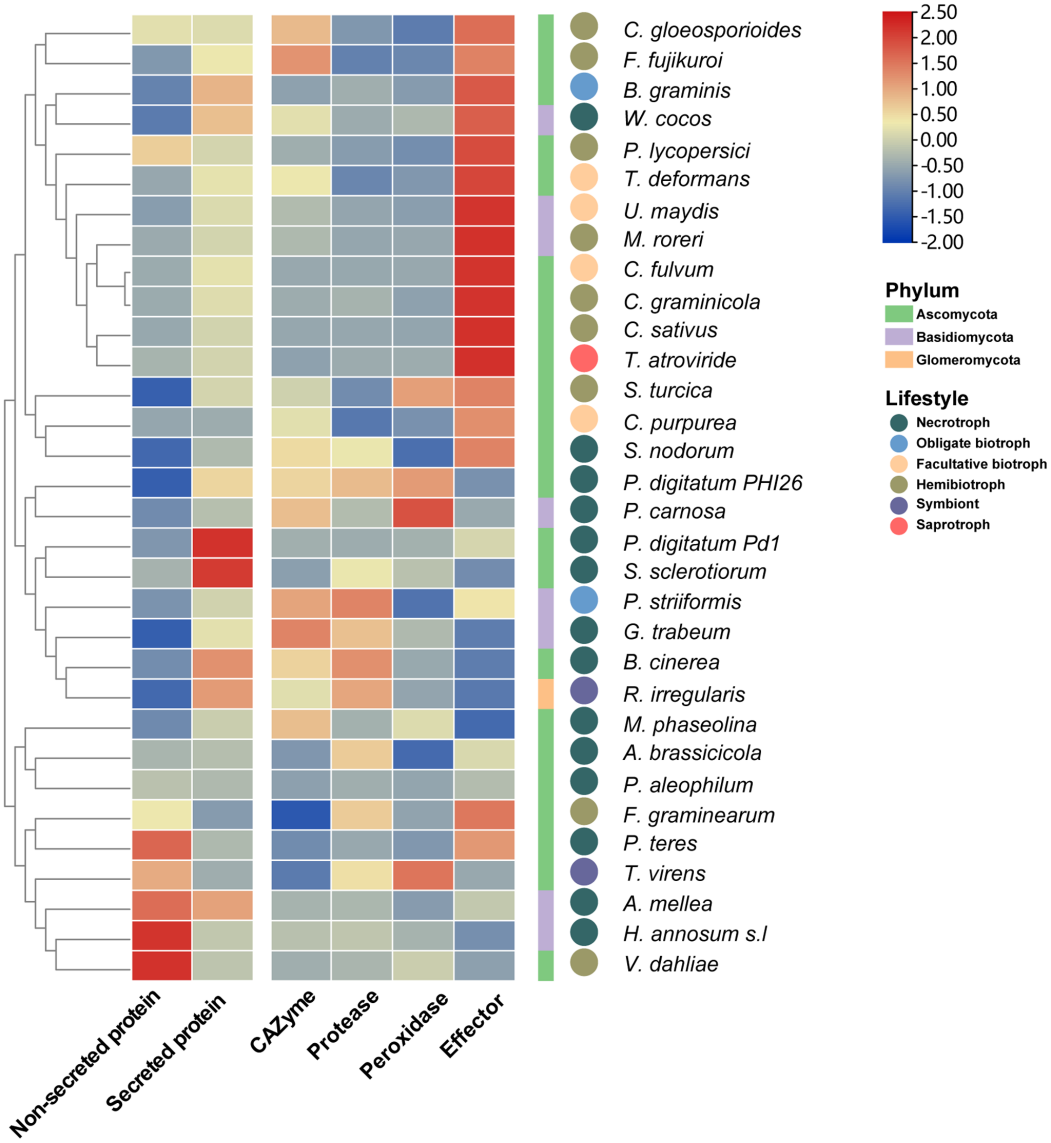
The heatmap of expression of SP genes in 32 fungi during plant infection. The heat map shows the average expression of non-SP genes, SP genes, CAZyme genes protease genes, peroxidase genes and effector genes during plant infection, the square legend represents the phylum to which the fungus belongs and the circle legend represents the fungal lifestyle.

To analyze the functions of fungal SPs during the infection processes, 32 sets of fungal-host interaction RNA-Seq data were collected from the NCBI SRA database (Supplementary Table S1). The results showed that the average expression level of genes encoding SPs (TPM = 150) was significantly higher (*p* = 0.0004) than that of genes encoding non-SPs (TPM = 73) in most fungi (Figure 7), indicating that the expression level of SPs was enhanced during the infection process. Furthermore, effector genes were found to be more highly expression in biotrophic and hemibiotrophic fungi than that in necrotrophic fungi. While genes encoding CAZymes, proteases and peroxidase were highly expressed in necrotrophic fungi. However, there were also some necrotrophic fungi whose effector expression levels were higher than those of secretases. For example, in the process of *Wolfiporia cocos* infection of spruce trees and *S. nodorum* infection of wheat, the expression level of effector genes was higher than that of secretase genes. We also found that different fungi with the same lifestyle had different expression patterns of SP genes with the same function when they infect their hosts. For example, the expression level of peroxidase genes was higher in *Trichoderma virens* infecting maize roots, whereas it was lower in *Rhizophagus irregularis* interacting with *Medicago truncatula*.

**Figure 8:**
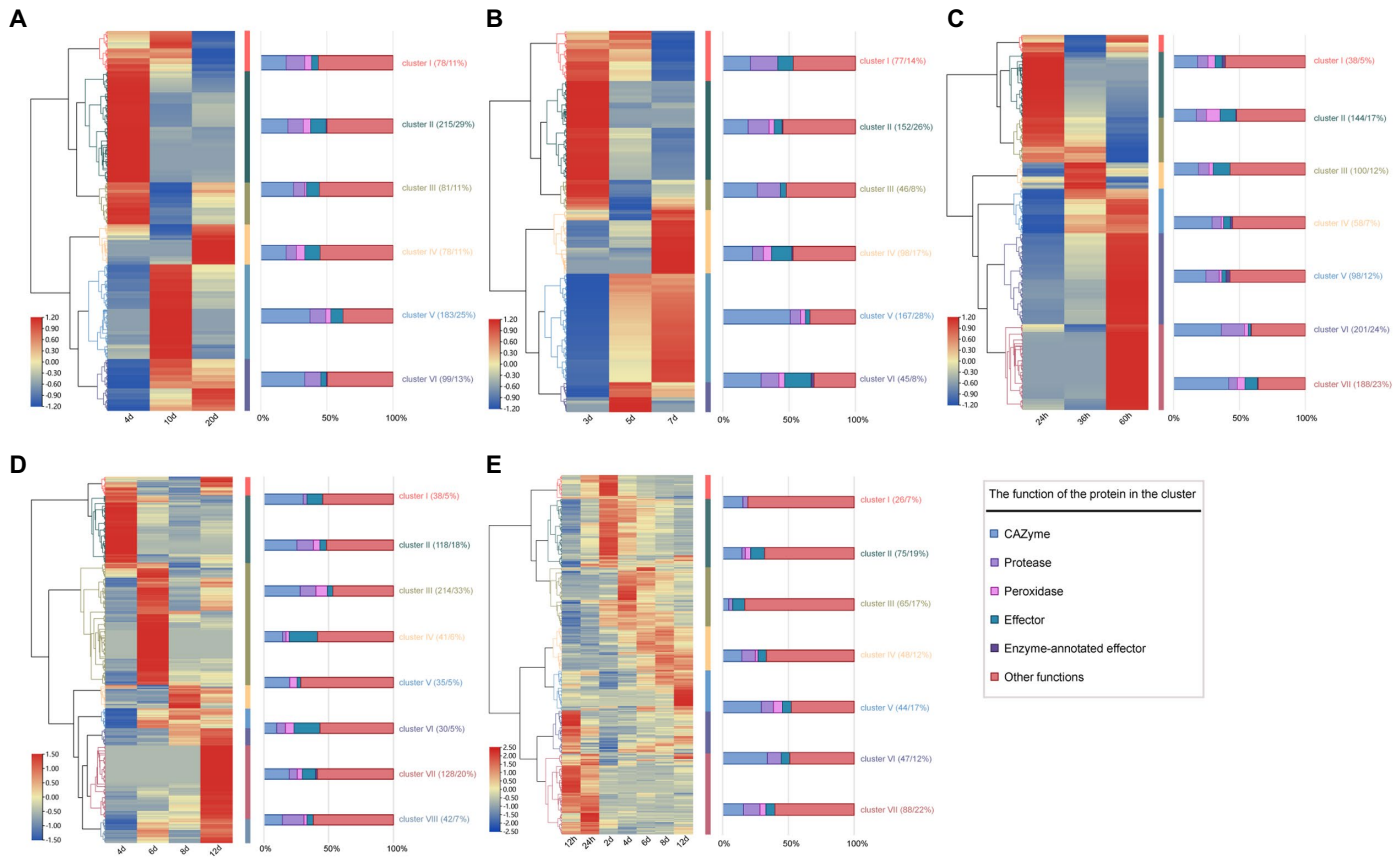
Heat map of expression of SP genes in hemibiotrophic and biotrophic fungi infecting plants. **(A)** Heat map of SP gene expression pattern in *F. fujikuroi* infecting maize at 4, 10, and 20  days. **(B)** Heat map of SP gene expression pattern in *F. graminearum* infecting maize at 3, 5, and 7  days. **(C)** Heat map of SP gene expression pattern in *C. graminicola* infecting *Arabidopsis thaliana* at 24, 36 and 60  h. **(D)** Heat map of SP gene expression in *Cladosporium fulvum* infected with tomato. **(E)** Heat map of SP gene expression in *Ustilago maydis* infected with tomato. The color change of the heat map indicates the level of SP gene expression at different infestation stages, the middle colored band indicates the cluster classification of the expression profile, and the right bar graph shows the proportion of SPs with different functions in each cluster.

Further, the expression pattern of SP genes in three hemibiotrophic (*Fusarium fujikuroi*, *F. graminearum* and *C. graminicola*) and two biotrophic fungi (*C. fulvum* and *Ustilago maydis*) was analyzed in detail. Cluster analysis of the expression profiles of three hemitrophic fungi found that the expression patterns of SP genes could be divided into 6 groups. The expression profiles of SP genes in *F. fujikuroi* and *F. graminearum* were very similar, nearly 40% of the genes were involved in the phases of penetration to biotroph fungal infection (cluster I and II of Figures 8A,B), and 50% of the genes were involved in the biotroph to necrotroph stages (cluster IV, V, and VI of Figures 8A,B). In *C. graminicola*, few SP genes were up-regulated during the stage of penetration to biotroph (cluster II, III, 29% of expressed genes), and 59% of expressed genes were highly expressed in the stage of biotroph to necrotroph (cluster IV, V, and VI; Figure 8C). Further, the expression patterns of SPs with different functions were studied. It was found that carbohydrases were the most expressed SPs when the hemibiotrophic fungi infested the host (Figures 8A–C) For example, it was found that the earliest activated SPs involved a smaller number of CAZymes, in which cellulases were predominant. As the infection progresses, more cellulase, pectin lyase and hemicellulase were upregulated in biotroph and necrotroph stages, especially hemicellulose, which was induced in 45 out of 62 hemicellulases of *F. graminearum*, 43 out of 57 of *F. fujikuroi* and 37 out of 50 of *C. graminicola*. This may be related to the fact that hemibiotrophic fungi acquire nutrients, destroy cell walls and release fungal spores in the middle and late stages of infection. In addition, the SP gene expression profiles of two biotrophic fungi, *C. fulvum* and *U. maydis*, were analyzed. It was found that biotrophic fungi tend to upregulate more protease and peroxidase genes in the early stage of infection than that in hemibiotrophic fungi, while CAZymes genes tend to be upregulated in the late stage of infection (Figures 8D,E). This may be related to the fact that biotrophic fungi need to obtain nutrients from host cells while maintaining the survival of host cells.

### Verification of secretory activity of the predicted signal peptide in *Setosphearia turcica*

**Figure 9:**
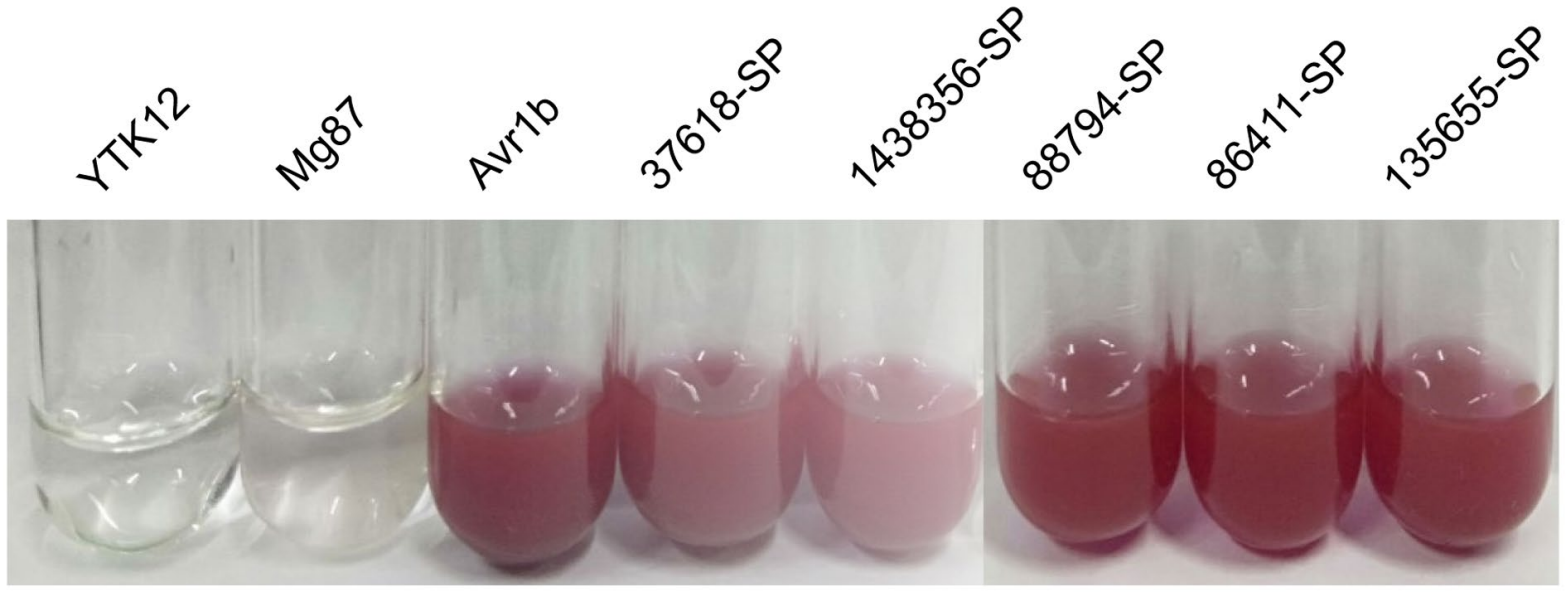
Verification of secretory function of signal peptide using the TTC staining assays.

To verify whether the predicted signal peptide has secretory activity, five predicted SPs (37,618, 1,438,356, 88,794, 86,411, and 135,655) from *S. turcica* were selected using the yeast strain YTK12 system. In addition, Avr1b, an effector in soybean blast that has a secretion function, was used as a positive control; Mg87, a functional signal peptide fragment that is not secreted in *M. oryzae*, was used as a negative control. The TTC staining assays showed that the positive control Avr1b and five signal peptides from *S. turcica* were able to secrete the invertase outside the cell, thereby degrading sucrose to monosaccharides, whereas neither the blank control YTK12 nor the negative control mg87 were able to do so (Figure 9), indicating that all the signal peptides of the five predicted SPs had secretory activity. This result further confirms the accuracy of our prediction of SPs.

## Discussion

Fungal secretome plays indispensable roles in nutrition acquisition and self-protection, which is important to explain the interaction mechanism between fungi and the environment. In recent years, the number of whole-genome sequencing of fungi has increased rapidly, which has extremely promoted the development of fungal genomics and laid the foundation for large-scale comparative analysis and study of the function of fungal SPs. In this study, we predicted the secretome of 150 fungi and explored their functions, which showed a preference between secretome and lifestyle in several aspects.

Currently, the most common methods for predicting SPs are to analyze the signal peptides and transmembrane domains. In addition, researchers typically analyze the subcellular locations and GPI sites of SPs to reduce false positives results for cell membrane proteins (Lowe and Howlett, 2012; Kim et al., 2016). To date, there are two publicly available fungal secretome databases, Fungal Secretome and Subcellular Proteome KnowledgeBase 2 (FunSecKB2) which was updated in 2013, and Fungal Secretome Database (FSD), which was created in 2010. FunSecKB2 uses six computational tools to make process-based predictions for 167 species of fungi (Meinken et al., 2014). The FSD has a very rich source of species, covering 452 fungal species, and the predicted results include non-classical SPs, but the screening conditions are relatively loose, mainly based on the signal peptide of SPs, which may contain a large number of false positives (Choi et al., 2010). In this study, we used the latest genomic information of each fungus and integrated the current mainstream software for predicting SPs. Our prediction results showed that each fungus contained an average of 633 SPs, accounting for 5.2% of total proteins, much less than the 15% of FSD. In the same 65 fungi predicted by present this study and FunSecKB2, we found that each fungus contained an average of 653 SPs, which is less than FunSecKB2 (an average of 710). This may due to the stringent parameters adopted in our process or the update of genomic information. For example, the two proteins (1,216,079 and 1,438,356) in the second version of the *S. turcica* genome were not predicted in FunSecKB2, but we successfully predicted them and verified them experimentally. Therefore, benefiting from the improvement of fungal genomic information and the optimization of the prediction software, our data can provide an important reference data for the study of fungal SPs.

By comparing the number of SPs, we found that hemibiotrophs and necrotrophs encode more SPs than biotrophs, saprotrophs, and symbionts. Compared with biotrophs, which depend on the viability of the host plant, non-biotrophic fungi require more secreted enzymes or toxins to kill the cells of the plant host and extract nutrients from the dead plant tissue. Thus, the largest secretome may be the basis for nutrient acquisition and manipulation of host plants by hemibiotrophs, as it has both biotrophic and necrotrophic phases (Rajarammohan, 2021). Furthermore, the CAZymes are involved in the degradation of plant cell walls, facilitating the process of pathogen infection and nutrient acquisition from the host (Ospina-Giraldo et al., 2010; Barrett et al., 2020). In our study, we found that the CAZymes were the most abundant predicted enzymes in the secretome. Hemibiotrophic fungi had the highest number of CAZymes, followed by necrotrophs, and the biotrophic fungi and symbiotic fungi had the lowest number of CAZymes. In addition, the hemibiotrophs and necrotrophs that eventually killed their plant hosts had similar CAZymes profiles and a more pronounced expansion of the GH, PL, CE and AA families. Furthermore, in the transcriptomic data, we found that genes encoding the cellulase of hemibiotrophic fungi were up-regulated early in the infection, which may be necessary to help the fungi penetrate the plant cell wall to achieve initial intracellular growth. As the infection processed, more and more CAZymes were up-regulated. For example, hemicellulases, which were highly up-regulated in the late stage of infection. However, in biotrophic fungi, CAZymes were not found to be up-regulated at a particular stage of infection, which may be related to the fact that biotrophic fungi need to obtain nutrients from host cells while maintaining host cell survival. These results indicates that hemibiotrophs and necrotrophs may require enhanced cell wall degradation during the infection process to complete the necrotrophic phase, but the symbionts and biotrophs weaken the ability to degrade the cell wall and it is possible that they act more on mycelial penetration of the plant epidermis or the pre-symbiotic stage. In addition, recent research has shown that ectomycorrhizal fungi secrete significantly lower average levels of CAZymes compared to plant pathogenic fungi (Lofgren et al., 2021). Powdery mildew can adapt to an obligate biotrophic lifestyle by contracting carbohydrate metabolism (Liang et al., 2018). These suggest that the amount and type of CAZymes in pathogens are closely related to their lifestyle.

Orthologs are genes in different species and often retain the same function during evolution (Koonin, 2005). By studying homologous groups, researchers can identify shared and specific genes among multiple species (Emms and Kelly, 2019). In the study on beneficial root endophyte *Colletotrichum tofieldiae* and its pathogenic relative *Colletotrichum incanum*, the author determined the genomic characteristics through the study of species-specific orthogroups and found that the transformation of the two *Colletotrichum* fungi only involves the reduction of effectors, and the expansion of chitin-binding and secondary metabolism-related protein families, indicating an evolutionary transition in *Colletotrichum* from pathogenic to symbiotic lifestyles (Hacquard et al., 2016). In the comparative genomics study of 23 species of *Aspergillus flavus*, it was found that highly conserved genes among species involved in aflatoxin biosynthesis, while species specific genes involved in encoding and regulating p450, which are relevant for fungal pathogenicity (Kjærbølling et al., 2020). In order to further explore the relationship between lifestyle and secretome, we clustered the secretome of 141 fungi and classified the orthogroups according to lifestyle to find shared and lifestyle-specific orthogroups, and the genes in the core orthogroups may play essential functions in supporting the survival of fungi. For example, the function of the core orthogroup, whose members come from all fungi, has been identified as serine carboxypeptidase, which has a variety of functions from catabolism to protein maturation. The functions of other highly conserved orthogroups were involved in metabolism, biological regulation, stress defense response, and pigment synthesis. For lifestyle-specific orthogroups, we found some lifestyle-adaptive functions. For example, in the specific orthogroups of symbiotic fungi, we found many hydrophobic surface binding A proteins, which transport fatty acids in host plants to fungi and establish a symbiotic nutrient exchange system with fatty acids as the primary carbon source (Jiang et al., 2018). We also found many CVHN domains in the symbiotic-specific orthogroups. These proteins bind to chitin oligomers and prevent them from functioning as pathogen-associated molecular patterns (PAMPs; Koharudin et al., 2015). In addition, we found many proteins annotated as flavin monooxygenase and carboxymuconolactone decarboxylase in the necrotrophic-specific orthogroups. Several studies have shown that flavin oxygenases attenuate the toxicity of phytoalexins and are involved in fungal cell wall growth and melanin deposition, and the carboxymuconolactone decarboxylase can promotes the expression of hydrolase and plays a crucial role in host pathogenesis (Wang et al., 2015; Pigné et al., 2017). In the hemibiotrophic-specific orthogroups, a large number of proteins have been identified as PAN domains for protein-oligosaccharide interactions, which increase the contact ability between fungi and host cells, thereby promoting fungal infection of the host (Yu et al., 2012). The biotrophic-specific orthogroups contain a large number of members, most of which are lifestyle-specific effectors without any functional annotations, which may be related to the long-term interaction between hemibiotrophic fungi and the host. Moreover, we believe that fungi with similar lifestyles share more orthogroups than fungi with more divergent lifestyles. Hemibiotrophic fungi and the necrotrophic fungi with a highly destructive necrotrophic stage in their life cycle share 158 orthogroups. Symbiotic fungi and biotrophic fungi that need to maintain the host survival share 33 orthogroups, which may indicate a conservative pattern of host cell penetration or recognition avoidance between biotrophs and symbionts. Saprophytic fungi and necrotrophic fungi share 45 orthogroups, which may reflect that necrotrophic fungi have an extended saprophytic stage as part of their life cycle (Laluk and Mengiste, 2010).

## Conclusion

In the present study, the secretome from 150 plant fungi was predicted and a total of 94,974 SPs were identified. Among them, 33,621 SPs were found to be functionally annotated, with their main functions being CAZymes, proteases, peroxidases and effectors, and the number of these proteins was closely related to their special lifestyles of the fungi. In addition, 1,513 orthogroups were identified. Among them, 1,416 were lifestyle-shared orthogroups which provided the most basic functions to support the lifecycle of fungus and 97 were lifestyle-specific orthogroups which were functionally related to the adaptation of the fungi to their specific lifestyles. The expression levels of these SP genes were higher than those of non-secreted protein genes and these SP genes in fungi with different lifestyles had their different expression patterns. Our research will contribute to understanding the changes in fungal lifestyle adaptations and provide new insights for future experiments to explore the mechanism of plant-fungus interaction.

## Data availability statement

The datasets analyzed in this study can be found in online repositories. The names of the repositories and accession numbers can be found in the article/Supplementary material.

## Author contributions

YL, SG, and JD conceived and designed the experiments. MJ and YL participated in the experiments and wrote the manuscript. MF, XG, HL, and HZ performed the experiments. All authors contributed to the article and approved the submitted version.

## Funding

This work was supported by the Hebei Provincial Central Leading Local Science and Technology Development Fund Project (216Z2902G), Basic Research Funds for Provincial Universities in Hebei Province (KY2022037 and KY2021042), and the China Agriculture Research System (CARS-02).

## Conflict of interest

The authors declare that the research was conducted in the absence of any commercial or financial relationships that could be construed as a potential conflict of interest.

## Publisher’s note

All claims expressed in this article are solely those of the authors and do not necessarily represent those of their affiliated organizations, or those of the publisher, the editors and the reviewers. Any product that may be evaluated in this article, or claim that may be made by its manufacturer, is not guaranteed or endorsed by the publisher.

## Footnotes

^1^http://bcb.unl.edu/dbCAN2/

^2^https://www.ebi.ac.uk/merops/

^3^http://peroxidase.riceblast.snu.ac.kr/

## References

[ref1] Almagro Armenteros J. J. Salvatore M. Emanuelsson O. Winther O. von Heijne G. Elofsson A.. (2019a). Detecting sequence signals in targeting peptides using deep learning. Life Sci Alliance 2:e201900429. doi: 10.26508/lsa.201900429, PMID: 31570514 PMC6769257

[ref2] Almagro Armenteros J. J. Tsirigos K. D. Sønderby C. K. Petersen T. N. Winther O. Brunak S.. (2019b). SignalP 5.0 improves signal peptide predictions using deep neural networks. Nat. Biotechnol. 37, 420–423. doi: 10.1038/s41587-019-0036-z, PMID: 30778233

[ref3] Altschul S. F. Gish W. Miller W. Myers E. W. Lipman D. J. (1990). Basic local alignment search tool. J. Mol. Biol. 215, 403–410. doi: 10.1016/S0022-2836(05)80360-2 2231712

[ref4] Barrett K. Jensen K. Meyer A. S. Frisvad J. C. Lange L. (2020). Fungal secretome profile categorization of CAZymes by function and family corresponds to fungal phylogeny and taxonomy: example Aspergillus and Penicillium. Sci. Rep. 10:5158. doi: 10.1038/s41598-020-61907-1, PMID: 32198418 PMC7083838

[ref5] Chen C. Chen H. Zhang Y. Thomas H. R. Frank M. H. He Y.. (2020). TBtools: an itegrative toolkit developed for interactive analyses of big biological data. Mol. Plant 13, 1194–1202. doi: 10.1016/j.molp.2020.06.009, PMID: 32585190

[ref6] Choi J. Détry N. Kim K. T. Asiegbu F. O. Valkonen J. P. T. Lee Y. H. (2014). fPoxDB: fungal peroxidase database for comparative genomics. BMC Microbiol. 14, 1–8. doi: 10.1186/1471-2180-14-117 PMC4029949 24885079

[ref7] Choi J. Park J. Kim D. Jung K. Kang S. Lee Y. H. (2010). Fungal secretome database: integrated platform for annotation of fungal secretomes. BMC Genomics 11:105. doi: 10.1186/1471-2164-11-105, PMID: 20146824 PMC2836287

[ref8] Doehlemann G. van der Linde K. Aßmann D. Schwammbach D. Hof A. Mohanty A.. (2009). Pep1, a secreted effector protein of Ustilago maydis, is required for successful invasion of plant cells. PLoS Pathog. 5:e1000290. doi: 10.1371/journal.ppat.1000290, PMID: 19197359 PMC2631132

[ref9] Eddy S. R. (2009). A new generation of homology search tools based on probabilistic inference. Genome Inform. 23, 205–211. PMID: doi: 10.1142/9781848165632_0019 20180275

[ref10] Emms D. M. Kelly S. (2019). OrthoFinder: phylogenetic orthology inference for comparative genomics. Genome Biol. 20:238. doi: 10.1186/s13059-019-1832-y, PMID: 31727128 PMC6857279

[ref11] Fang A. Han Y. Zhang N. Zhang M. Liu L. Li S.. (2016). Identification and characterization of plant cell death–inducing secreted proteins from Ustilaginoidea virens. Mol. Plant Microbe Interact. 29, 405–416. doi: 10.1094/MPMI-09-15-0200-R, PMID: 26927000

[ref12] Hacquard S. Kracher B. Hiruma K. Münch P. C. Garrido-Oter R. Thon M. R.. (2016). Survival trade-offs in plant roots during colonization by closely related beneficial and pathogenic fungi. Nat. Commun. 7:11362. doi: 10.1038/ncomms11362, PMID: 27150427 PMC4859067

[ref13] He Q. Liu Y. Liang P. Liao X. Li X. Li X.. (2021). A novel chorismate mutase from Erysiphe quercicola performs dual functions of synthesizing amino acids and inhibiting plant salicylic acid synthesis. Microbiol. Res. 242:126599. doi: 10.1016/j.micres.2020.126599, PMID: 33010586

[ref14] Hemetsberger C. Herrberger C. Zechmann B. Hillmer M. Doehlemann G. (2012). The Ustilago maydis effector Pep1 suppresses plant immunity by inhibition of host peroxidase activity. PLoS Pathog. 8:e1002684. doi: 10.1371/journal.ppat.1002684, PMID: 22589719 PMC3349748

[ref15] Horton P. Park K. J. Obayashi T. Fujita N. Harada H. Adams-Collier C. J.. (2007). WoLF PSORT: protein localization predictor. Nucleic Acids Res. 35, 585–587. doi: 10.1093/nar/gkm259 PMC1933216 17517783

[ref16] Jacobs K. A. Collins-Racie L. A. Colbert M. Duckett M. Golden-Fleet M. Kelleher K.. (1997). A genetic selection for isolating cDNAs encoding secreted proteins. Gene 198, 289–296. doi: 10.1016/S0378-1119(97)00330-2 9370294

[ref17] Jiang Y. Xie Q. Wang W. Yang J. Zhang X. Yu N.. (2018). Medicago AP2-domain transcription factor WRI5a is a master regulator of lipid biosynthesis and transfer during mycorrhizal symbiosis. Mol. Plant 11, 1344–1359. doi: 10.1016/j.molp.2018.09.006, PMID: 30292683

[ref18] Jones J. D. G. Dangl J. L. (2006). The plant immune system. Nature 444, 323–329. doi: 10.1038/nature05286 17108957

[ref19] Käll L. Krogh A. Sonnhammer E. L. (2007). Advantages of combined transmembrane topology and signal peptide prediction--the Phobius web server. Nucleic Acids Res. 35, 429–432. doi: 10.1093/nar/gkm256 PMC1933244 17483518

[ref20] Kim K. T. Jeon J. Choi J. Cheong K. Song H. Choi G.. (2016). Kingdom-wide analysis of fungal small secreted proteins (SSPs) reveals their potential role in host association. Front. Plant Sci. 7:186. doi: 10.3389/fpls.2016.00186 26925088 PMC4759460

[ref21] Kjærbølling I. Vesth T. Frisvad J. C. Nybo J. L. Theobald S. Kildgaard S.. (2020). A comparative genomics study of 23 Aspergillus species from section Flavi. Nat. Commun. 11:1106. doi: 10.1038/s41467-019-14051-y, PMID: 32107379 PMC7046712

[ref22] Koharudin L. M. Debiec K. T. Gronenborn A. M. (2015). Structural insight into fungal cell wall recognition by a CVNH protein with a single LysM domain. Structure 23, 2143–2154. doi: 10.1016/j.str.2015.07.023, PMID: 26455798 PMC4635050

[ref23] Koonin E. V. (2005). Orthologs, paralogs, and evolutionary genomics. Annu. Rev. Genet. 39, 309–338. doi: 10.1146/annurev.genet.39.073003.114725 16285863

[ref24] Krogh A. Larsson B. von Heijne G. Sonnhammer E. L. (2001). Predicting transmembrane protein topology with a hidden markov model: application to complete genomes. J. Mol. Biol. 305, 567–580. doi: 10.1006/jmbi.2000.4315, PMID: 11152613

[ref25] Laluk K. Mengiste T. (2010). Necrotroph attacks on plants: wanton destruction or covert extortion? Arabidop Book 8:e0136. doi: 10.1199/tab.0136, PMID: 22303261 PMC3244965

[ref26] Liang P. Liu S. Xu F. Jiang S. Yan J. He Q.. (2018). Powdery mildews are characterized by contracted carbohydrate metabolism and diverse effectors to adapt to obligate biotrophic lifestyle. Front. Microbiol. 9:3160. doi: 10.3389/fmicb.2018.03160, PMID: 30619222 PMC6305591

[ref27] Lo Presti L. Lanver D. Schweizer G. Tanaka S. Liang L. Tollot M.. (2015). Fungal effectors and plant susceptibility. Annu. Rev. Plant Biol. 66, 513–545. doi: 10.1146/annurev-arplant-043014-114623 25923844

[ref28] Lofgren L. A. Nguyen N. H. Vilgalys R. Ruytinx J. Liao H. L. Branco S.. (2021). Comparative genomics reveals dynamic genome evolution in host specialist ectomycorrhizal fungi. New Phytol. 230, 774–792. doi: 10.1111/nph.17160, PMID: 33355923 PMC7969408

[ref29] Lowe R. Howlett B. (2012). Indifferent, affectionate, or deceitful: lifestyles and secretomes of fungi. PLoS Pathog. 8:e1002515. doi: 10.1371/journal.ppat.1002515, PMID: 22396640 PMC3291654

[ref30] McCotter S. W. Horianopoulos L. C. Kronstad J. W. (2016). Regulation of the fungal secretome. Curr. Genet. 62, 533–545. doi: 10.1007/s00294-016-0578-2 26879194

[ref31] Meinken J. Asch D. K. Neizer-Ashun K. A. Chang G. Cooper C. Min X. J. (2014). FunSecKB2: a fungal protein subcellular location knowledgebase. Comput Mol Biol 4, 1–17. doi: 10.5376/cmb.2014.04.0007

[ref32] Mir A. A. Park S. Y. Sadat M. A. Kim S. Choi J. Jeon J.. (2015). Systematic characterization of the peroxidase gene family provides new insights into fungal pathogenicity in *Magnaporthe oryzae*. Sci. Rep. 5:11831. doi: 10.1038/srep11831, PMID: 26134974 PMC4488832

[ref33] Morais Do Amaral A. Antoniw J. Rudd J. J. Hammond-Kosack K. E. (2012). Defining the predicted protein secretome of the fungal wheat leaf pathogen *Mycosphaerella graminicola*. PLoS One 7, –e49904. doi: 10.1371/journal.pone.0049904 PMC3517617 23236356

[ref34] Muszewska A. Stepniewska-Dziubinska M. M. Steczkiewicz K. Pawlowska J. Dziedzic A. Ginalski K. (2017). Fungal lifestyle reflected in serine protease repertoire. Sci. Rep. 7:9147. doi: 10.1038/s41598-017-09644-w, PMID: 28831173 PMC5567314

[ref35] Ospina-Giraldo M. D. Griffith J. G. Laird E. W. Mingora C. (2010). The CAZyome of Phytophthora spp.: a comprehensive analysis of the gene complement coding for carbohydrate-active enzymes in species of the genus Phytophthora. BMC Genomics 11:525. doi: 10.1186/1471-2164-11-525, PMID: 20920201 PMC2997016

[ref36] Pantazopoulou A. Glick B. S. (2019). A kinetic view of membrane traffic pathways can transcend the classical view of Golgi compartments. Front. Cell Dev. Biol. 7:153. doi: 10.3389/fcell.2019.00153, PMID: 31448274 PMC6691344

[ref37] Pertea M. Kim D. Pertea G. M. Leek J. T. Salzberg S. L. (2016). Transcript-level expression analysis of RNA-seq experiments with HISAT, StringTie and Ballgown. Nat. Protoc. 11, 1650–1667. doi: 10.1038/nprot.2016.095, PMID: 27560171 PMC5032908

[ref38] Pigné S. Zykwinska A. Janod E. Cuenot S. Kerkoud M. Raulo R.. (2017). A flavoprotein supports cell wall properties in the necrotrophic fungus Alternaria brassicicola. Fungal Biol Biotechnol 4, 1–13. doi: 10.1186/s40694-016-0029-3, PMID: 28955470 PMC5611651

[ref39] Quevillon E. Silventoinen V. Pillai S. Harte N. Mulder N. Apweiler R.. (2005). InterProScan: protein domains identifier. Nucleic Acids Res. 33, 116–120. doi: 10.1093/nar/gki442 PMC1160203 15980438

[ref40] Rajarammohan S. (2021). Redefining plant-necrotroph interactions: the thin line between hemibiotrophs and necrotrophs. Front. Microbiol. 12:673518. doi: 10.3389/fmicb.2021.673518, PMID: 33995337 PMC8113614

[ref41] Rapoport T. A. Li L. Park E. (2017). Structural and mechanistic insights into protein translocation. Annu. Rev. Cell Dev. Biol. 33, 369–390. doi: 10.1146/annurev-cellbio-100616-060439 28564553

[ref42] Sigrist C. J. A. Cerutti L. de Castro E. Langendijk-Genevaux P. S. Bulliard V. Bairoch A.. (2010). PROSITE, a protein domain database for functional characterization and annotation. Nucleic Acids Res. 38, 161–166. doi: 10.1093/nar/gkp885 PMC2808866 19858104

[ref43] Skamnioti P. Gurr S. J. (2007). *Magnaporthe grisea* Cutinase2 mediates appressorium differentiation and host penetration and is required for full virulence. Plant Cell 19, 2674–2689. doi: 10.1105/tpc.107.051219, PMID: 17704215 PMC2002628

[ref44] Sperschneider J. Dodds P. N. Gardiner D. M. Singh K. B. Taylor J. M. (2018). Improved prediction of fungal effector proteins from secretomes with EffectorP 2.0. Mol. Plant Pathol. 19, 2094–2110. doi: 10.1111/mpp.12682, PMID: 29569316 PMC6638006

[ref45] Sperschneider J. Gardiner D. M. Dodds P. N. Tini F. Covarelli L. Singh K. B.. (2016). EffectorP: predicting fungal effector proteins from secretomes using machine learning. New Phytol. 210, 743–761. doi: 10.1111/nph.13794, PMID: 26680733

[ref46] Wang J. Y. Zhou L. Chen B. Sun S. Zhang W. Li M.. (2015). A functional 4-hydroxybenzoate degradation pathway in the phytopathogen Xanthomonas campestris is required for full pathogenicity. Sci. Rep. 5:18456. doi: 10.1038/srep18456, PMID: 26672484 PMC4682078

[ref47] Yu Y. Jiang D. Xie J. Cheng J. Li G. Yi X.. (2012). Ss-Sl2, a novel cell wall protein with PAN modules, is essential for sclerotial development and cellular integrity of Sclerotinia sclerotiorum. PLoS One 7:e34962. doi: 10.1371/journal.pone.0034962, PMID: 22558105 PMC3338822

