# Supplementary figures and images for: Identification and analysis of the secretome of plant pathogenic fungi reveals lifestyle adaptation
Source: Front Microbiol. 2023 Apr 20;14:1171618. doi: 10.3389/fmicb.2023.1171618 (PMC10156984; doi:10.3389/fmicb.2023.1171618)

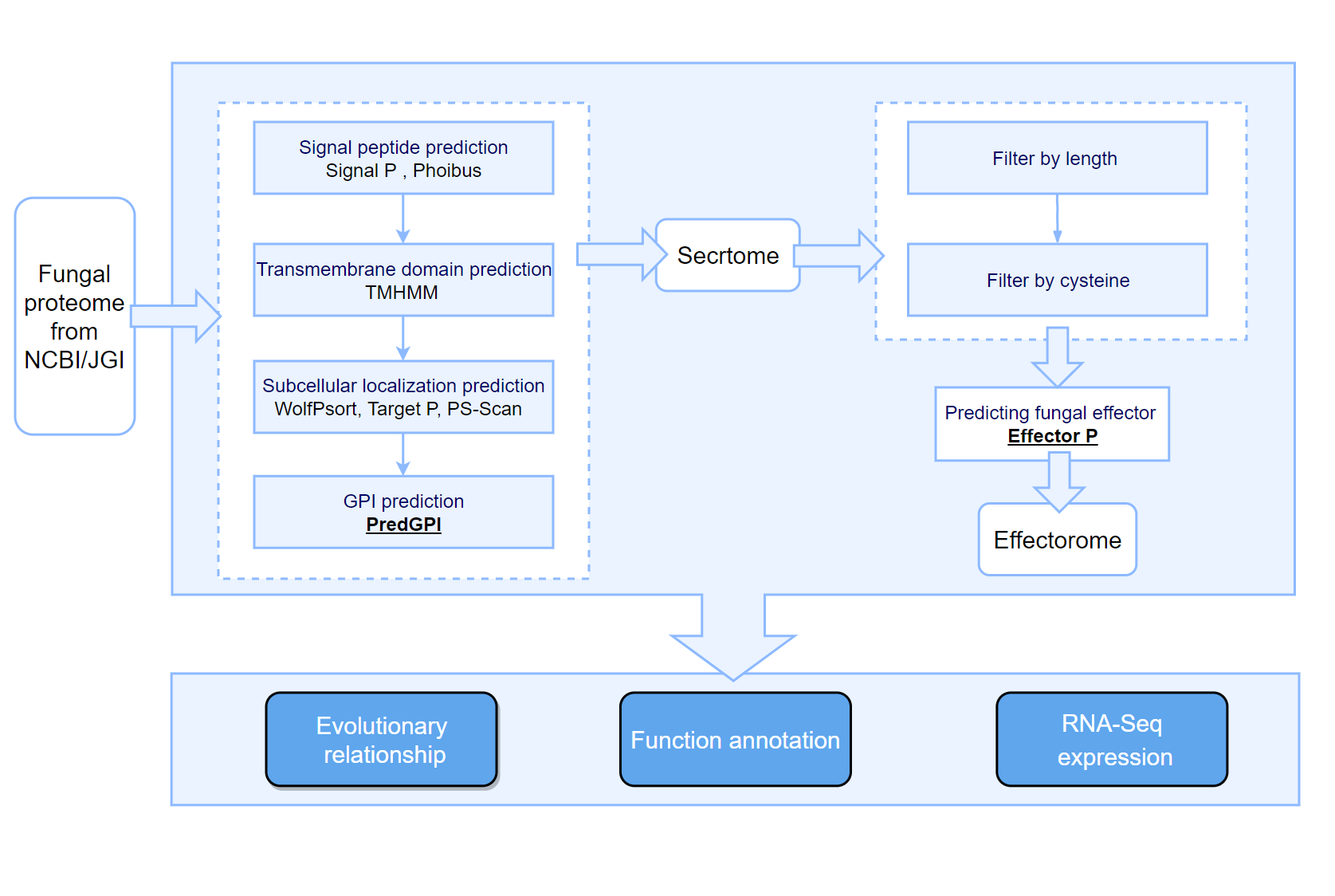

Supplement: Supplementary file 1 [file Image_1.TIF]

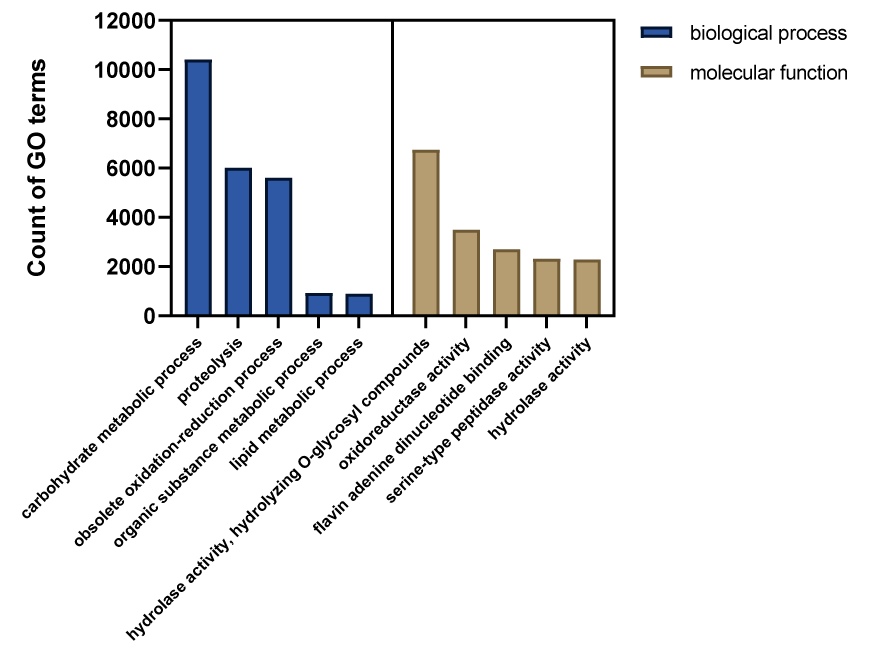

Supplement: Supplementary file 2 [file Image_2.TIF]
